# An historical overview of artificial intelligence for diagnosis of major depressive disorder

**DOI:** 10.3389/fpsyt.2024.1417253

**Published:** 2024-11-13

**Authors:** Hao Liu, Hairong Wu, Zhongli Yang, Zhiyong Ren, Yijuan Dong, Guanghua Zhang, Ming D. Li

**Affiliations:** ^1^ State Key Laboratory for Diagnosis and Treatment of Infectious Diseases, The First Affiliated Hospital, Zhejiang University School of Medicine, Hangzhou, China; ^2^ Shanxi Tongchuang Technology Inc., Taiyuan, China; ^3^ Shanxi Province Mental Health Center, Taiyuan Psychiatric Hospital, Taiyuan, China; ^4^ Shanxi Yingkang Healthcare General Hospital, Yuncheng, Shanxi, China; ^5^ School of Big Data Intelligent Diagnosis and Treatment Industry, Taiyuan University, Taiyuan, China

**Keywords:** major depressive disorder, MDD, artificial intelligence, large language model, AI agent, multimodal diagnosis

## Abstract

The Artificial Intelligence (AI) technology holds immense potential in the realm of automated diagnosis for Major Depressive Disorder (MDD), yet it is not without potential shortcomings. This paper systematically reviews the research progresses of integrating AI technology with depression diagnosis and provides a comprehensive analysis of existing research findings. In this context, we observe that the knowledge-driven first-generation of depression diagnosis methods could only address deterministic issues in structured information, with the selection of depression-related features directly influencing identification outcomes. The data-driven second-generation of depression diagnosis methods achieved automatic learning of features but required substantial high-quality clinical data, and the results were often obtained solely from the black-box models which lack sufficient explainability. In an effort to overcome the limitations of the preceding approaches, the third-generation of depression diagnosis methods combined the strengths of knowledge-driven and data-driven approaches. Through the fusion of information, the diagnostic accuracy is greatly enhanced, but the interpretability remains relatively weak. In order to enhance interpretability and introduce diagnostic criteria, this paper offers a new approach using Large Language Models (LLMs) as AI agents for assisting the depression diagnosis. Finally, we also discuss the potential advantages and challenges associated with this approach. This newly proposed innovative approach has the potential to offer new perspectives and solutions in the diagnosis of depression.

## 1 Introduction

Depression is a prevalent and chronic mental illness that significantly impacts an individual’s cognition, emotions, and behaviors (1, 2). With the rapid development of modern society and the increasing pressures of work and life, the global prevalence of depression has reached 4.4%, making it one of the most common and severe mental disorders worldwide (3). MDD affects as many as 12% of adults globally, with the highest prevalence in the United States observed among the youth, women, and elderly (4). In China, the lifetime prevalence of severe depression is 3.4%, with a 12-month prevalence of 2.1% (5). In the context of the escalating incidence of depression, clinical diagnosis primarily relies on scale screening and psychiatric interviews. However, the diagnostic outcomes of this method depend on the clinician’s experience and the patient’s cooperation, leading to a high rate of misdiagnosis. The Value of Treatment study conducted by the European Brain Council suggested that approximately 52% of MDD episodes went undiagnosed, and about 38% of diagnosed patients did not receive treatment (6). Consequently, there is an urgent need for an objective, efficient, accurate, and convenient diagnostic method for depression.

With the rapid advancement of AI technology, a substantial body of researches has explored the application of AI in assisting the diagnosis and prediction of depression. The first generation of knowledge-driven approaches for depression diagnosis primarily relied on classical Machine Learning (ML) techniques for prediction. These approaches involved the extraction of features from various sources, such as speech signals, facial expressions, and Electroencephalogram (EEG) data, by using feature extraction algorithms. ML models were then constructed based on these extracted features for depression prediction and diagnosis (7, 8). However, the feature extraction process often requires supposed prior knowledge and the loss of information can be inevitable. The research trend has since shifted from traditional handcrafted feature design towards more advanced methods that can automatically learn features. With the increase of clinical data related to depression and the development of Deep Learning (DL) techniques, more and more approaches based on DL models have been applied to depression diagnosis, marking the birth of data-driven second-generation depression diagnostic approaches. DL models, designed end-to-end, have the capability to automatically learn features during model training. They could encode and classify data without relying on feature extractors which designed in advance, thereby automating the process of depression diagnosis (9, 10). However, DL networks remain black-box models, often lacking interpretability. For reasons of safety in the medical industry, relying solely on the diagnostic results generated by DL networks can be challenging for clinical application. The core idea behind the third generation of depression diagnostic methods is to combine the strengths of the previous two generations to enhance diagnostic accuracy. By integrating manually extracted features with those represented by DL models and employing various multimodal feature fusion strategies, a more comprehensive and accurate diagnosis of depression can be achieved. The combination of manually extracted features with complex models can better uncover depressive feature information, while information fusion, by combining heterogeneous information from different sources, ensures high-quality information processing for more comprehensive and reliable decision-making (11). Nevertheless, the third-generation diagnostic approaches still lack of interpretability which are critical in clinical practices. Additionally, while they can significantly improve the diagnostic accuracy, they tend to complicate the overall diagnostic process, moving away from the goal of the fully automated depression diagnosis.

With the proliferation of data and computational power available for training AI models, models like GPT-4 (Generative Pre-trained Transformer 4) (12, 13), Llama (14, 15), and GLM (General Language Model) (16, 17), among others, have achieved significant success. These LLM models exhibit strong generalizability and emergent abilities. Researchers have begun exploring their potential applications in the medical field, including medical knowledge-based question answering, medical X-ray interpretation, and medical treatment recommendations. Given the versatility and efficiency of LLMs and their highly interpretable text-based outputs, combining the goal of automating depression diagnosis with LLMs thus represents a new direction in the realm of depression diagnostic assistance.

In this review, we conducted a systematic literature search across PubMed, IEEE Xplore, and Google Scholar, covering the period from January 2010 to April 2024. The search used combinations of keywords such as “Artificial Intelligence”, “Major Depressive Disorder”, “MDD”, “depression”, “diagnosis”, “machine learning” and “deep learning”. We only included peer-reviewed studies published in English that focused on the application of AI to the diagnosis of MDD and provided detailed methodologies and quantitative results. We excluded those non-peer-reviewed works, studies lacking methodological clarity or quantitative outcomes, or studies focused solely on treatment. Articles were initially screened by title and abstract, followed by a full-text review. A total of 114 studies were selected based on relevance, methodological rigor, and their contribution to the field, ensuring that key advancements and gaps in AI-based depression diagnosis were thoroughly addressed.

## 2 Scale-based evaluation methods

The assessment of depression using scale-based approaches primarily includes self-assessment of scales and physician assessment according to clinical diagnostic criteria (**Table 1**). Commonly recognized diagnostic criteria include International Classification of Diseases, 11th (ICD-11) (18), Diagnostic and Statistical Manual of Mental Disorders, 5th Edition (DSM-V) (19, 20), and Mini-International Neuropsychiatric Interview (MINI) (21, 22). Other commonly used clinical scales still include Patient Health Questionaire-9 Items (PHQ-9) (23, 24), the Self-Rating Anxiety Scale (SAS) (25, 26), and the Self-Rating Depression Scale (SDS) (27, 28).

**Table 1:** Clinical diagnostic criteria and related scales for depression.

Function	Scale	Pattern	Scale Description
Clinical diagnostic criteria	DSM-V	Others-rating	It’s a comprehensive guide for diagnosing mental disorders, featuring standardized criteria essential for clinical practice, research, and education in mental health.
	ICD-11	Others-rating	ICD-11 is global classification system by WHO for diseases and health conditions, aiding diagnosis and documentation worldwide.
	MINI	Others-rating	The MINI is a brief psychiatric diagnostic interview used by mental health professionals, covering various disorders efficiently and thoroughly.
Depression scales	PHQ-9	Self-rating	PHQ-9 is a self-administered questionnaire used as a screening tool for depression. It consists of nine questions based on the Diagnostic and Statistical DSM-V criteria for major depressive disorder. PHQ-9 scores help assess the severity of depression symptoms.
	HAMD-17	Others-rating	HAMD-17 is a clinician-administered questionnaire used to assess the severity of depression symptoms in individuals. It comprises 17 items covering various aspects of depression, such as mood, guilt, suicidal ideation, and sleep disturbances.
	SDS	Self-rating	SDS is a self-report depression screening tool developed. It consists of 20 items designed to measure the level of depression in individuals.
	MARDS	Others-rating	MADRS is a clinician-administered tool comprising 10 items to assess depression severity. It evaluates symptoms like sadness, tension, sleep problems, and concentration difficulties, aiding clinicians in monitoring depression and treatment response.
	BDI	Self-rating	BDI is a self-report questionnaire assessing depression severity in adults and adolescents. With 21 items covering mood, guilt, and physical symptoms, it yields scores indicating the severity of depressive symptoms.

DSM-V, Diagnostic and Statistical Manual of Mental Disorders; ICD-11, International Classification of Diseases; MINI, Mini-International Neuropsychiatric Interview; PHQ-9, Patient Health Questionaire-9 Items; HAMD-17, Hamilton Depression Scale; SDS, Self-rating Depression Scale; MARDS, Montgomery-Asberg Depression Rating Scale; BDI, Beck depression rating scale.

The PHQ-9 is currently the most used self-rating scale, where users evaluate their behavior and total scores over the past two weeks based on scoring criteria. The Hamilton Depression Scale-17 Items (HAMD-17) (29) scale is one of the most commonly used ones, which requires a combination of two professional physicians to evaluate patients and independently score them. Generally, a combination of conversation and observation is used. Among them, the self-rating scale is relatively simple and convenient, but the answer of users to the scale question solely based on personal self-awareness is more subjective. The other-rating scales used in clinics is relatively time-consuming and laborious, but more professional and accurate. In evaluating depression scales, Wu et al. (30) utilized the Hospital Anxiety and Depression Scale-Depression subscale (HADS-D) to screen for depression. Their results demonstrated that HADS-D exhibited varying sensitivity and specificity depending on the type of diagnostic interview employed. Among 101 eligible studies, which included 25,574 participants with 2,549 diagnosed with MDD, it was found that for semi-structured interviews, fully structured interviews, and the MINI, the optimal cut-off value for HADS-D was 7 or higher, yielding a sensitivity of 0.82 and a specificity of 0.78. As the cut-off value increased, specificity improved, but sensitivity decreased; for instance, at a cut-off value of 11, sensitivity was 0.44, and specificity was 0.95 [Wu et al. (30)]. These findings indicate that HADS-D’s performance varies across different diagnostic contexts, and the choice of cut-off value significantly influences the accuracy of screening results. Thus, this suggests that, in clinical practice, different cut-off values should be selected on the basis of specific needs of patient populations in order to balance the risks of false positives and false negatives, thereby more effectively identifying patients with depression.

## 3 Automated diagnosis approaches combining with AI

Diagnostic results based on traditional scale-based methods rely heavily on the experience of clinicians and patient cooperation, which could lead to a high rate of misdiagnosis. Additionally, limited healthcare resources imply that diagnostic services are probably available to only a small portion of the population. In contrast, AI technology can automate the analysis of respondents’ linguistic content and facial expressions, reducing reliance on self-reported data. This approach enhances the objectivity of the assessment, decreases the likelihood of misdiagnosis, and expands the accessibility of diagnostic services.

With the advancement of diagnostic and therapeutic techniques for depression, an increasing volume of research related to depression has generated a substantial amount of clinical diagnostic and treatment data. AI technology, with its inherent strengths in handling large datasets, multidimensional information, and multimodal data holds great promise in this context (31, 32). Therefore, leveraging the automatic learning capabilities of AI to depression diagnosis represents an effective method towards automating the diagnosis of depression (33, 34). In the following sections, we will provide a description of each generation of AI technology in terms of their research and applications on depression.

### 3.1 The first-generation diagnostic methods for depression

The depression diagnostic model, constructed using traditional ML algorithms, is referred to as the first-generation depression diagnostic method. This method relied on robust prior knowledge and necessitated the use of various feature extraction algorithms to identify depression-related features. Subsequently, ML models with relatively simple structures were built based on these features. This enabled the model to automatically learn from the feature data and optimize its performance, ultimately achieving the goal of assisting in depression prediction.

#### 3.1.1 Text features methods

The linguistic characteristics of depressed patients often manifest as pessimism, low self-esteem, and unclear, repetitive, or incoherent speech content (35). Currently, classification methods on text contents can be broadly categorized into grammar rules combined with statistical approaches and ML methods.

Grammar rules combined with statistical methods typically involve the manual construction of domain-specific sentiment lexicons. During the sentiment recognition process, a comparison of the words in the input text with the domain-specific sentiment lexicon or calculates vector distances is commonly used to identify matching sentiment words and their polarities. In the context of constructing Chinese depression sentiment lexicons, Yin et al. (36) proposed a sentiment word mining method that combines word frequency statistics and sentiment word intensity. Liu et al. (37) expanded upon a basic sentiment lexicon by using the Term Frequency–Inverse Document Frequency (TF-IDF) algorithm to select domain-specific sentiment lexicons and extend them through word similarity calculations. When using sentiment lexicons for text classification, He et al. (38) introduced a PRE-TF-IDF classification algorithm based on weight preprocessing. This method enhances text classification accuracy by adding weight preprocessing and word density weighting to the TF-IDF algorithm.

For traditional ML approaches, the primary method involves manually extracting features from sample datasets and selecting suitable classification models based on these features. The choice of an appropriate classifier based on text features is crucial for text sentiment classification, and commonly used classifiers include Support Vector Machine (SVM), Naive Bayesian (NB), Extreme Gradient Boosting (XGBoost), K-Nearest Neighbors (KNN), among others. Zhang et al. (39) conducted a comparative study on the prediction of depression diagnosis using different ML algorithms based on NHANES data. The results showed that SVM and CatBoost classifiers performed best in the total feature set, while they had the lowest performance in the dietary feature set. From a data application perspective, Diao et al. (40) systematically analyzed several reports on ML-based text classification and concluded that SVM is frequently used in medical datasets due to its ability to effectively overcome challenges such as high-dimensional calculations and overfitting.

#### 3.1.2 Speech features methods

Through clinical observation and statistics, individuals with depression typically show the following characteristics in their speech behavior: slow speech rate, low voice intensity, reduced spontaneity, and unclear articulation, among others (41). These speech behavioral characteristics can be manifested through various speech features, including prosodic features such as pitch, intonation, energy, and rhythm variations, as well as fundamental frequency features resulting from vocal cord vibrations, and speech rate features that express the speed of speech. It is known that individuals with depression usually exhibited a faster decline in speech rate and longer response times (42). There existed a negative correlation between pitch and loudness with the severity of depression (43). Shankayi et al. (44) also found that a combination of features, including prosody, voice spectrum, and glottal features, yields better recognition results than any single feature type. In the realm of audio feature sets, Geneva Minimalistic Acoustic Parameter Set (eGeMAPS) (45) and COVAREP are widely recognized feature sets. eGeMAPS is often used as an acoustic feature set in depression detection challenges like the audio/visual emotion challenge and workshop (AVEC) (46).

Sumali et al. (47) collected data from 300 depression patients and used the Least Absolute Shrinkage and Selection Operator (LASSO) algorithm to perform feature selection on the participants’ audio data to predict depression using a linear SVM model. They achieved a classification accuracy of up to 93.3% on their test set. Cummins et al. (48) employed Gaussian Mixture Models (GMMs) to model depressive and neutral speech, using Mel Frequency Cepstral Coefficient (MFCC) features from audio as the primary discriminative feature. They tested their approach on a dataset of 23 depression patients and 24 control subjects, achieving a classification accuracy of 82%. Jiang et al. (49) analyzed the classification performance of prosodic, spectral, and glottal speech features in depression recognition and proposed an ensemble logistic regression model, ELRDD-E, for detecting depression in speech. In an automatic depression classification task on a dataset of 170 Chinese native subjects, the accuracy rate for females was 75.00%.

#### 3.1.3 Visual features methods

The mental state of individuals with depression is correlated with changes in their facial expressions. Common features observed in the visual analysis of their faces include a lack of expression, a somber demeanor, a forlorn look, and evasive eye contact (1). For instance, there are statistically significant differences in eye movement patterns between depression patients and healthy individuals (50). Depression is often associated with greater downward gaze angles, shorter average gaze durations, and reduced smile intensity (51). In studies related to psychomotor disturbances in bipolar disorder, depression patients exhibit longer reaction times in gaze tasks (52). In the domain of facial feature extraction tools, the OpenFace package (53) stands as a cutting-edge open-source tool for facial motion analysis. This tool provides functionalities such as facial landmark detection, head pose tracking, eye gaze tracking, and facial action unit recognition.

Wang et al. (54) extracted key facial features from collected facial videos using a human activity appearance model. By using SVM for depression classification based on variations in eye, eyebrow, and mouth movements, the authors achieved an accuracy of 78.85%, a recall rate of 80.77%, and an F1-score of 0.792 in a sample with 26 subjects. Li et al. (55) extracted facial features, including eyes, eyebrows, mouth, and nose, and utilized an average performance of three classification models: SVM, NB, and Random Forest (RF), for predicting depression in both male and female patients, which achieved an average classification accuracy of 71.5% for females and 66.7% for males. Additionally, their results indicated that eyebrows and mouth contribute more to neutral emotional valence than other facial areas. Li and Fan (56) conducted a review of facial feature studies in depression patients from the year of 2016 to 2021 using ML approach. They concluded that SVM, NB, RF are the most commonly used classifiers in facial feature research of depression patients. Further, they pointed out that many studies overlook the temporal aspects of emotional changes in depression patients and suggested that multimodal fusion approach is an important future research direction, particularly in the context of limited clinical sample size and relatively narrow research objectives (57).

#### 3.1.4 Other modal features methods

In addition to the research on behavioral features in depression patients, researchers have also explored various other modalities, including brain imaging, electrophysiological data, and omics data for studying depression. In the first-generation research on brain imaging for depression diagnosis, Bhaumik et al. (58) utilized features from the left posterior cingulate cortex and the right posterior dorsolateral prefrontal cortex. They employed the SVM algorithm to distinguish severe depression patients from healthy controls, achieving an accuracy of 76.1%. Wei et al. (59) extracted resting-state networks and calculated their Hurst index using range-scale analysis. They achieved a recognition rate of 90% for depression patients using a linear kernel SVM. In research on electrophysiological data for assisting depression diagnosis, Akbari et al. (60) proposed a method for detecting depression using brainwave signal reconstruction in phase space and geometric features. The framework, with features selected using particle swarm optimization and a support vector machine classifier, achieved an average classification accuracy of 99.30% and a Matthews Correlation Coefficient (MCC) of 0.98. In the domain of omics data, Dipnall et al. (61) employed ML enhanced regression algorithms and logistic regression to identify three key biomarkers related to depression: red cell distribution width, serum glucose, and total bilirubin.

#### 3.1.5 Multimodal features methods

Differences in feature data for depression patients manifest across multiple modalities, and the information from these modalities complements each other. Therefore, the development of depression diagnosis methods that combine multi-modal data features is an important research direction for achieving automated diagnostic assistance.

Yang et al. (62) combined audio, video, language, and sleep data features and used a decision tree model to distinguish between normal individuals and depression patients. They tested their approach on the Distress Analysis Interview Corpus of human and computer interviews (DAIC) dataset (63), achieving an average F1-score of 0.724. Alghowinem et al. (64) fused audio features, gaze features, and head features of participants and optimized an SVM classifier using Library for Support Vector Machines (LibSVM) (65). Subsequently, in a real clinical validation dataset consisting of 30 patients with severe depression and 30 healthy controls, the accuracy of single-modal classification results was 83% for audio, 73% for gaze, and 63% for head features. The fused multimodal approach achieved an average accuracy of 88%, effectively demonstrating the complementary nature of features across different modalities. Zhao et al. (66) combined speech and facial visual data to propose a multi-modal fusion depression diagnosis algorithm. They introduced spectrum subtraction, orthogonal matching pursuit algorithms, and a cascade based on the ratio of sound and facial emotion. Their approach achieved a diagnostic accuracy of 81.14%, which was 6.76% higher than using only audio as a single modality.

#### 3.1.6 Summary of the first-generation diagnostic methods for depression

**Figure 1:**
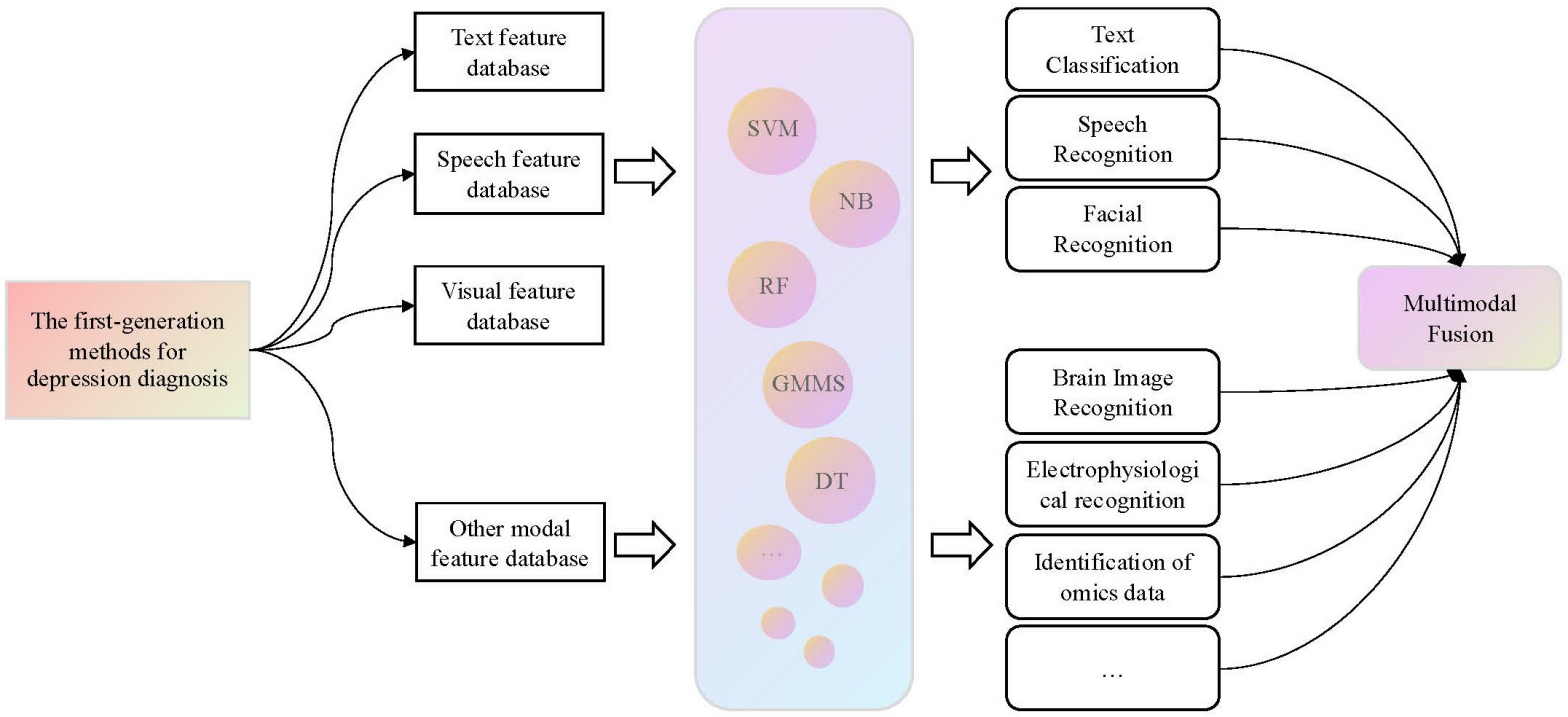
The first-generation framework for depression diagnosis methods. For this generation of methods, it is necessary to first extract modal features related to depression from the raw data, and then use classical ML algorithms to classify these features to identify patients with depression.

The first-generation AI depression diagnosis methods relied on prior knowledge and traditional ML algorithms to identify depression patients. These methods exhibited strong interpretability. In these approaches, the feature selection in ML directly influenced the best performance of the identification results. Therefore, it is essential to extract a sufficient number of depression-related features in order to effectively improve the recognition accuracy. Our current understanding of depression is considered to be limited, which could lead to partial data presentation and, to some extent, restrict the effectiveness of model classification. Consequently, the extraction of a sufficient number of depression-related features formed the foundation for the quality of these methods. Furthermore, the process of manually extracting prior knowledge from raw data was often inefficient. Therefore, the research trend in AI-based depression diagnosis methods has shifted from traditional handcrafted feature design to more advanced methods that involve automatic feature learning. The first-generation framework for depression diagnosis methods is summarized in **Figure 1**.

### 3.2 The second-generation diagnostic methods for depression

Inspired by the brain’s neural system, specific approaches based on DL models have been successfully applied in the realm of sentiment analysis. One such application is the development of a depression diagnostic model, termed the second-generation depression auxiliary diagnostic method. The primary problem it addressed was how to automate the extraction of various modality feature data from respondents and train a diagnostic model based on a large volume of feature data. In this section, we provide a comprehensive overview of existing second-generation diagnostic methods, categorizing them broadly based on the types of data employed into textual, audio, video, other type, and multimodal data-driven depression auxiliary diagnostic methods.

#### 3.2.1 Text features methods

In the context of depression recognition research that combines social network text data with DL, Xu (67) proposed a depression analysis model based on an attention mechanism. This model takes into account the characteristics of social text data, employs automatic feature selection techniques, and utilizes a multi-instance learning approach. Ive et al. (68) introduced an Recurrent Neural Network (RNN) architecture that incorporates an attention mechanism to predict potential depression patients. This model can effectively extract essential textual elements and make accurate predictions, due to its integration of the attention mechanism. Coppersmith et al. (69) conducted research on the presence of suicide attempts within social network text. They used Long Short-Term Memory (LSTM) networks to quantify textual signals related to suicide attempts and captured contextual information between textual contents, thus obtained text content associated with suicide.

#### 3.2.2 Speech features methods

In research focused on diagnostic methods based on acoustic features, Chlasta et al. (70) introduced an ensemble learning approach based on Convolutional Neural Networks (CNN), which exhibited promising performance on the AVEC2016 dataset (71). Lu et al. (72) addressed the issues of complex model structures and low recognition rates associated with Deep Neural Network (DNN) methods and proposed a model that combines residual thinking and attention mechanisms, achieving an accuracy rate of 76%. Li and Fu (73) conducted research from both feature design and network architecture perspectives by introducing a Multi-scale Audio Differential Normalization (MADN) feature extraction algorithm and developed a depression recognition regression model called DR AudioNet based on this algorithm.

#### 3.2.3 Visual features methods

Depression diagnostic methods that combine DL techniques with facial visual features can generally be categorized into two major approaches. One is the “Visual Local” approach, which is based on previous research on facial features associated with depression. In this approach, specific local features related to depression are identified and used as inputs to DL models. This method requires fewer features and tends to converge more easily during model training. The other is the “Visual Global” approach, which aims to prevent information loss during the feature extraction process by treating the entire facial information as input to the model for automated feature extraction. However, this method requires extensive training on a large amount of video data and the construction of complex DL models.

In the “Visual Local” research, feature extraction methods primarily involve using visual extraction tools, integrated systems, or various devices such as eye-tracking devices to directly extract feature information from specific facial regions. These features include changes in facial landmark points, the frequency of muscle unit occurrences, eye gaze angles, and more. For example, Wang et al. (54) achieved a recognition rate of 78% by utilizing features extracted from eye movements, eyebrow movements, and pupil motions obtained during interviews, combined with other modal features. Gao (74) built a depression recognition model based on multi-scale facial features using facial Action Units (AUs) and facial landmark points and demonstrated that multi-scale features outperformed single-scale features.

In “Visual Global” research, the entire video is typically used as input to the model for automatic feature extraction. Xu (75) addressed the varying impact of different facial regions on the recognition of depression and constructed the dual pooling residual attention network (DPRAN), incorporating a residual attention mechanism. This network was used to extract spatiotemporal features from facial images to complete the depression recognition task. De Melo et al. (76) explored global features, local features, and spatiotemporal correlations of facial regions and proposed a depression recognition architecture based on 3D convolutional neural networks.

#### 3.2.4 Other modal features methods

In the research on depression auxiliary diagnosis methods based on EEG, Acharya et al. (77) employed a CNN model for depression screening on the basis of EEG signals. This method allows for automatic adaptive learning from input EEG signals to differentiate between EEG signals of individuals with and without depression. The algorithm achieved recognition accuracies of 93.5% and 96.0% for left and right hemisphere EEG signals, respectively, in a sample size of 30 subjects. In the study of predicting antidepressant response to severe depression using genetic biomarkers, Lin et al. (78) developed a DL predictive method, with the goal of predicting the possible antidepressant treatment outcomes for MDD using 10 Single Nucleotide Polymorphism (SNP) data associated with antidepressant response. The predictions were made using a Multi-layer Feedforward Neural Network (MFNN) model with two hidden layers, achieving the Area Under the ROC Curve (AUC) of 0.8228 and sensitivity of 0.7546.

#### 3.2.5 Multimodal features methods

**Figure 2:**
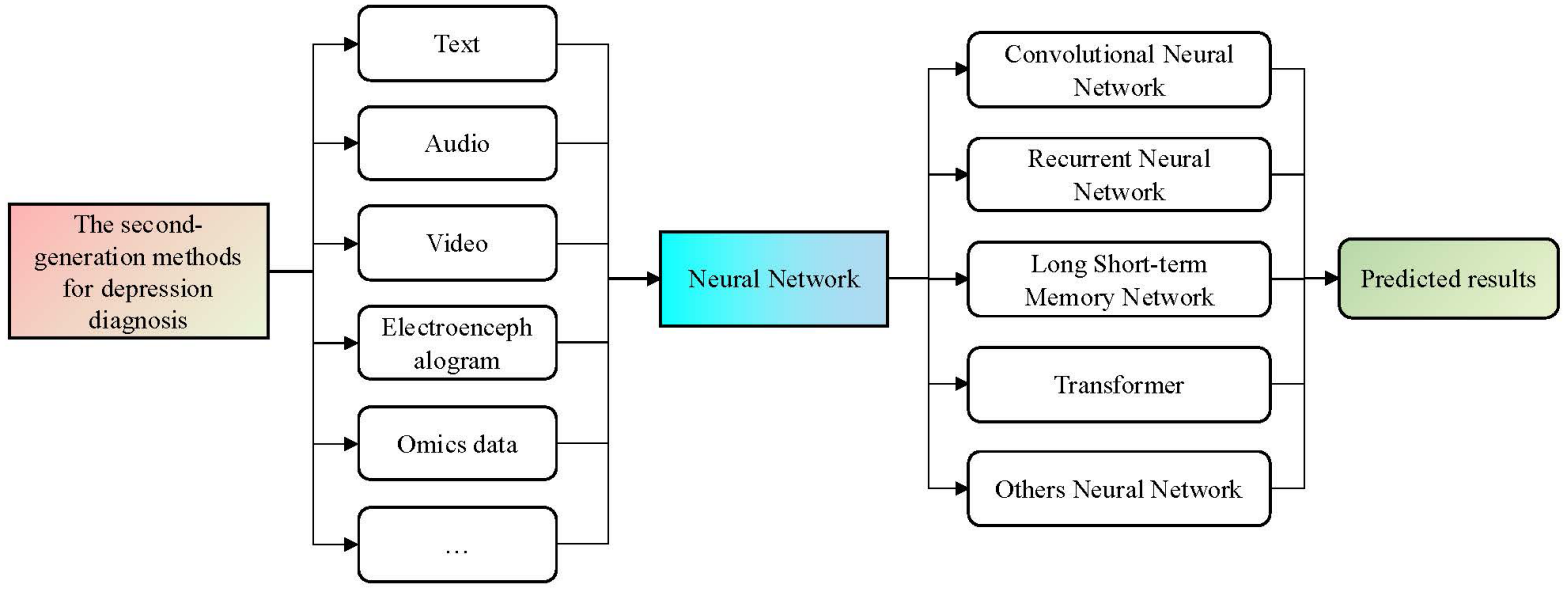
The second-generation framework for depression diagnosis methods. This generation of methods typically utilize the trained DL models on large-scale datasets as tools to represent different modal features. By using the end-to-end design and model training of neural networks, the diagnosis process of depression can be realized automatically.

Previous research has indicated that methods based on single-modal features such as text content, audio signals, and facial visual cues have produced promising results in depression diagnosis. However, the behavioral characteristics of individuals with depression manifest in multiple modalities, and the information across these modalities is complementary. Results from AVEC (46) have consistently demonstrated that, compared to single-modal depression recognition methods, multimodal fusion methods tend to yield a better performance. The framework for second-generation depression diagnostic methods is depicted in **Figure 2**.

In the realm of research on multimodal fusion methods in second-generation diagnostic approaches, Wang et al. (79) summarized nine statistical features related to user text, social behavior, and images as well. They extracted deep representations of text using the XLNet model and integrated them with other modal data to construct a Multimodal Feature Fusion Network (MFFN). This method achieved an F1-score of 0.9685 on the WU3D test dataset. Hao (80) employed Chinese Bidirectional Encoder Representations from Transformers (ChineseBERT), ResNet101 V2, and Bidirectional Long Short-Term Memory (BiLSTM) models to extract features from text, visual information, and audio data, respectively, and used an attention algorithm to fuse these features and effectively calculated the complementarity and contribution of multimodal information by continuously adjusting the weights of each modality.

#### 3.2.6 Summary of the second-generation diagnostic methods for depression

The second-generation depression diagnostic methods typically utilize different DL models trained on large-scale datasets as tools for modality feature representation. In contrast to first-generation diagnostic methods, they do not require prior knowledge and circumvent the complex process of manual feature extraction. Through an end-to-end design, these methods can automatically learn feature extraction and achieve depression diagnosis (9, 81). However, up to this point, researchers are still unable to explain the inner workings of DL networks. With DL networks, they can only obtain diagnostic results without insight into the basis for the diagnosis. Since DL networks are considered to black-box models, relying solely on the diagnostic results from deep networks may pose challenges in clinical applications, especially concerning medical industry safety-related issues.

### 3.3 The third-generation diagnostic methods for depression

**Figure 3:**
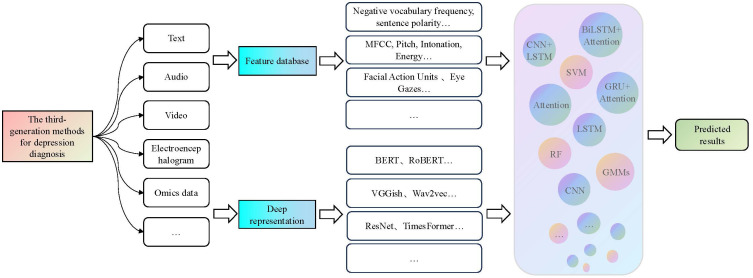
The third-generation framework for depression diagnosis methods. This generation of methods first combine prior features with depth features and then use a modal fusion strategy to integrate multiple model features as inputs for machine learning and neural network models used in the recognition of depression.

The third-generation diagnostic method for depression combines the advantages of the first and second-generation methods, aiming to improve the accuracy of depression diagnosis. It utilizes manually extracted depression features as prior knowledge and also employs deep learning models to capture deep representation information from different modalities of features. Finally, it integrates multiple sources of information through various fusion strategies to achieve depression recognition. In terms of fusion paradigm, data-level fusion, feature-level fusion, and decision-level fusion are the main directions explored in related studies (82). The framework of the third-generation diagnostic method for depression is illustrated in **Figure 3**.

#### 3.3.1 The data-level fusion methods

**Figure 4:**
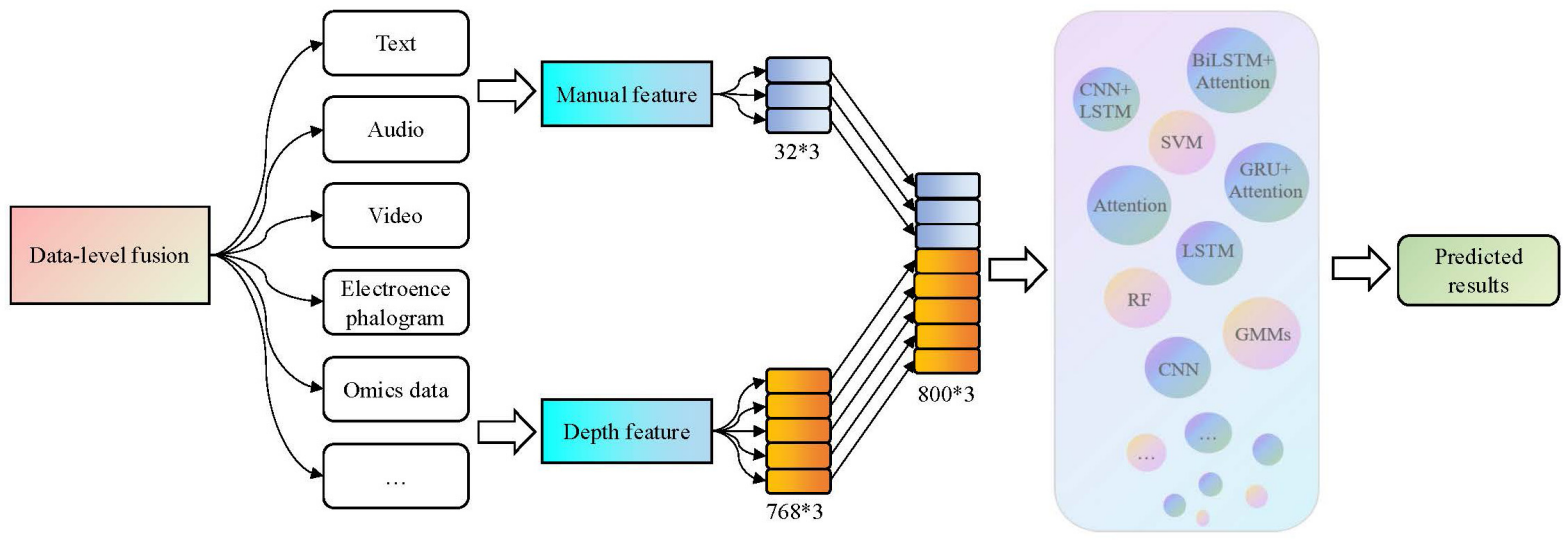
The data-level fusion process in the third-generation depression diagnosis methods. By using a simple feature vector concatenation method, varied features from different modalities and dimensions can be concentrated into comprehensive features and then input them into a neural network for depression recognition.

Data-level fusion, also known as early fusion, is an easily implementable method. In data-level fusion methods, different modalities of data are concatenated into integrated features using simple feature vectors, which are then fed into a neural network for depression recognition (83). The data-level fusion framework is shown in **Figure 4**.

In the research of data-level fusion, Zou et al. (84) extracted the 768-dimensional textual deep representation of interviewees based on interview content using the BERT model. They also extracted the 12-dimensional visual features and 25-dimensional audio features of interviewees using the Openface and eGemaps tools. Finally, they achieved automated identification of depression based on the Bi-LSTM model, with a recall rate of 0.89 and the F1-score of 0.88 on the Chinese Multimodal Depression Corpus (CMDC) dataset. Semwal et al. (85) collected over 10 million publicly available Twitter posts and approximately 10,000 images and obtained the feature vector representations of text and images using the Doc2Vec model and EfficientNetV2S architecture, respectively. The final accuracy of this approach reached 99.3%, with an F1 score of 95.61%. This was 17.36% higher than the best text model and 35.99% higher than the best image model, demonstrating that the information contained in text and images is complementary and can be effectively combined.

Although the data-level fusion method is relatively simple, it is a successful technique that combines features from different modalities for depression recognition. Compared to single-modality feature recognition methods, it considers more comprehensive information and enhances the reliability of diagnostic results. However, one drawback of data-level fusion is the high dimensionality of the combined feature vector. Considering this drawback, Joshi et al. (86) proposed data fusion and Principal Component Analysis (PCA) after data fusion, retaining 98% of the variance. The results showed that training a depression detection model on the dimensionality-reduced feature set can also improve system performance.

#### 3.3.2 The feature-level fusion methods

**Figure 5:**
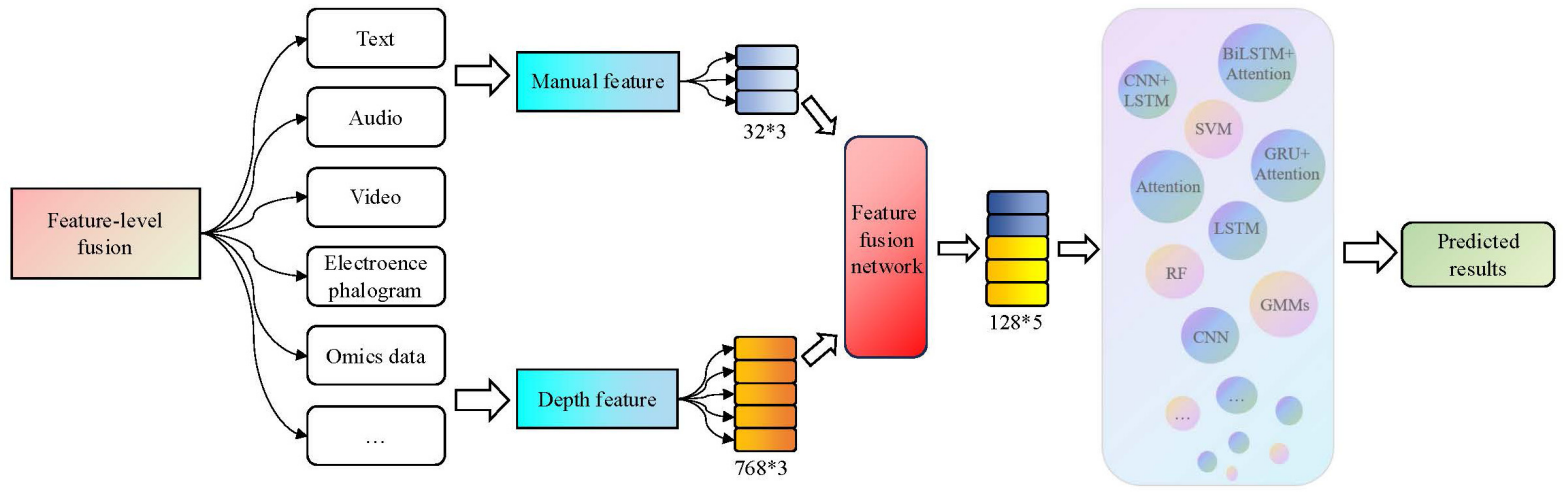
The feature-level fusion process in the third-generation depression diagnosis methods. By adding a feature fusion network, emotional features extracted from different modalities and dimensions are combined to form a comprehensive emotional feature.

Fusion at the feature-level, also known as intermediate fusion, combines the emotion features extracted from different modalities in a certain way to form a comprehensive emotion feature as the input to identify networks. The fusion methods typically include neural networks, attention mechanisms, residual networks, and their combinations. The framework for feature-level fusion is illustrated in **Figure 5**.

In the research of feature-level fusion, Bucur et al. (87) proposed a method for identifying users with depression on social media platforms by combining text, image, and temporal information. They utilized EmoBERTa and Contrastive Language–Image Pre-training (CLIP) to encode the text and image, processed the encoded image and text sequences using a cross-modal encoder, and finally used a transformer encoder along with the relative posting time encoded into position embeddings to identify users with depression. Haque et al. (88) employed Causal Convolutional Neural Networks (C-CNN) to transform a variable-length sequence summarizing long-term auditory, visual, and linguistic elements into a fixed-size numerical vector, which was used to fuse the textual, audio, and 3D video features of facial landmark points. The model demonstrated promising performance on the DAIC-WOZ dataset.

Compared to the concatenation approach of feature vector fusion at the data-level, feature-level fusion utilizes intermediate fusion networks to transform and fuse different modality data, thus enhancing the correlation and complementary effects among the data. The effectiveness of the fusion network directly affects the diagnostic accuracy, but the specific fusion network required for different modal combinations or data may vary. Therefore, many researchers have begun investigating unified modality fusion encoders, such as the Visual-Image Encoder (89) and the Meta-Transformer (90) that fuses 12 modalities of data.

#### 3.3.3 The decision-level fusion methods

**Figure 6:**
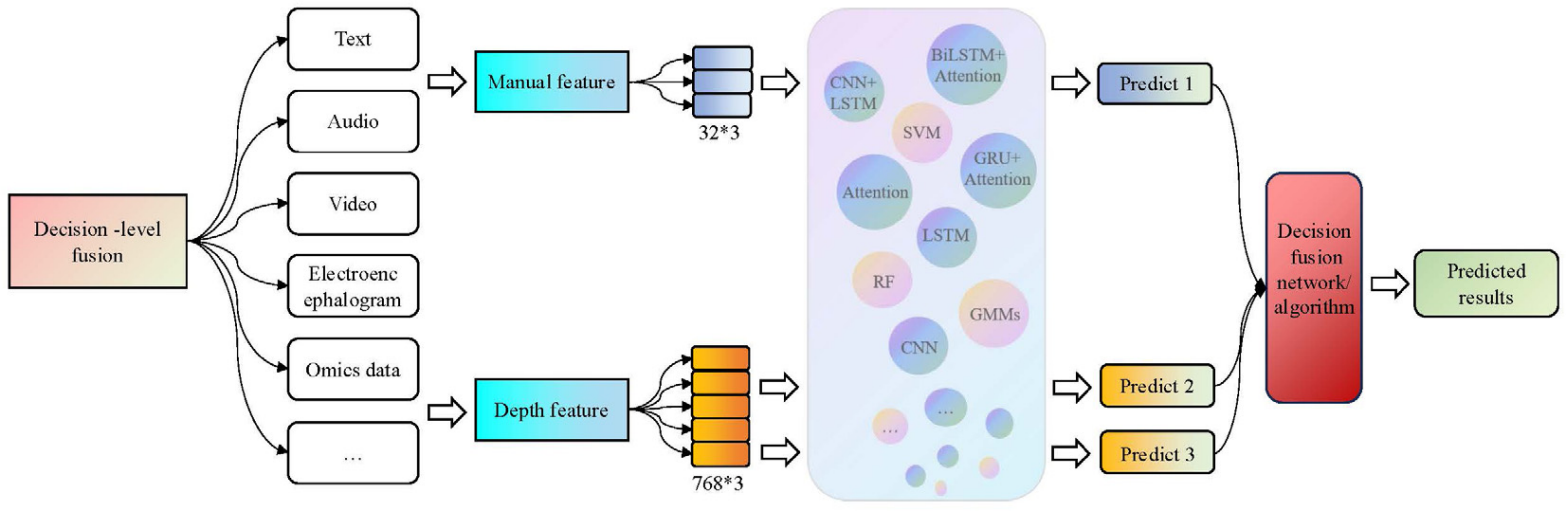
The decision-level fusion process in the third-generation depression diagnosis methods. It constructs strong feature extraction models and classifiers for each modality, and then selects appropriate fusion strategies to obtain the final results.

Decision-level fusion is also known as late-fusion. Firstly, it constructs strong feature extraction models and classifiers for each modality and then selects appropriate fusion strategies to obtain the final results. Common fusion strategies include voting, weighting, and ML algorithms. The decision-level fusion framework is shown in **Figure 6**.

In terms of decision-level fusion research, Zhang et al. (91) proposed a deep forest based multimodal data fusion method for depression recognition to fuse multimodal data such as Histogram of Oriented Gradients (HOG), landmark, and AU of video frames. Zou et al. (84) integrated text content, acoustic features, and facial visual features, and implemented automatic alignment of multimodal features and depression diagnosis based on the Multiple Transformers (MulT) model. Yang et al. (82) proposed a multimodal fusion framework consisting of Deep Convolutional Neural Network (DCNN) and DNN models. For each mode, manually created feature descriptors are input into DCNN to learn advanced global features with compact dynamic information, and then the learned features are fed to DNN to predict the PHQ-8 score, with the final Mean Absolute Error (MAE) of the model of 5.163.

Decision-level fusion constructs a strong single modal feature extraction model based on the characteristics of each modality, which can effectively capture the internal information of each modality and help improve recognition rate. But one challenge is that decision-level fusion relies heavily on the selection of fusion algorithms, and the results are easily affected and interfered with by certain single modal information.

#### 3.3.4 Other fusion methods

In addition to the methods of data-level fusion, feature-level fusion, and decision-level fusion, methods such as hybrid fusion, multi-level cascading, and a combination of multiple methods are currently also important research directions. For example, Wei et al. (92) introduced a sub attention model ConvBiLSTM that connects heterogeneous information based on the method of decision-level fusion. After incorporating this module, the diagnostic results of the model always outperform traditional decision-level fusion methods. Ray et al. (93) proposed a new multimodal depression prediction network based on multi-level attention networks, which integrates features of audio, video, and text modalities while learning correlations within and between modalities. The optimal performance was achieved in AVEC2019 (46), which was 17.52% higher than the baseline at that time.

#### 3.3.5 Summary of the third-generation diagnostic methods for depression

The third-generation diagnostic methods combine the advantages of the previous two generations. The manually extracted features and DL model features have different attributes. Combining manual features with deep features can not only utilize previous experience but also mine hidden information in the original data. Secondly, the information of different modalities has complementarity, and through the strategy of modal fusion, feature information can be more comprehensively integrated to improve the accuracy of diagnostic results. However, the third-generation diagnostic methods still belong to the black box model and have weak interpretability. Although manual features are combined, their role in the diagnostic process cannot be explained. Secondly, while improving the accuracy of diagnosis, the strategy of feature combination and modal fusion also makes the entire diagnosis process more complex and far away from end-to-end goals.

### 3.4 Summary and analysis of diagnostic methods for the previous three generations

In view of the shortcomings of scale-based assessment, the method combined with AI technology has made great progress in diagnosis efficiency and objectivity, and a large number of experiments have also verified that the diagnosis effect of multimode is generally better than that of single mode, as shown in **Table 2**. From years of research in depression diagnosis and current clinical practices, language contents and facial expressions are commonly recognized as the modalities that are most closely related to depressive symptoms. In the future, AI models for depression diagnosis will integrate more types of data and knowledge, and thus interpret them more precisely. By incorporating various data sources that have been proven to correlate with depression, such as physiological signals and long-term behavioral information, AI models can more comprehensively capture the complex characteristics of depression.

**Table 2:** Comparison of results between single mode and multimodal fusion methods.

Method	Dataset	Measurement	Modality	Results
Anshul et al. (94)	Covid-19	Recall	T+S+D+U	0.78
			V+T+D+U	0.76
			V+T+S+U	0.84
			V+T+S+D	0.87
			V+S+D+U	0.89
			V+T+S+D+U	**0.91**
Zhang et al. (95)	Private	Recall	A	0.61
			V	0.64
			T	0.64
			A+V	**0.78**
Suri et al. (85)	Private	Accuracy	T	0.82
			I	0.63
			T+I	**0.99**
Bucur et al. (87)	Twitter (96)	Accuracy	T	0.87
			I	–
			T+I	**0.93**
Zou et al. (84)	CMDC	Recall	A	0.83
			V	0.71
			T	**0.90**
			A+V+T	0.89
Wei et al. (92)	DAIC-WOZ	Accuracy	A	0.77
			V	0.80
			T	–
			A+V+T	**0.83**
Ahmed et al. (97)		Recall	T+A	0.89
			T+E+F	0.92
			A+F	0.91
			T+A+E+F	**0.93**
Our proposed third-generation method	Private	Accuracy	A	0.64
			V	0.51
			T	0.73
			A+V+T	**0.78**

This table lists the major datasets used in AI research for diagnosing depression. The quality of the selected datasets was assessed based on their availability, representativeness of the studies, and relevance to the topic of this review. For example, the DAIC-WOZ dataset is a representative public dataset in the field of depression recognition. It has undergone ethical review, includes detailed annotations, and has been widely used in the AVEC series of competitions, demonstrating its reliability and broad applicability in this field. For private datasets, we ensured their usability by consulting the relevant references to verify the credibility of the data sources and the validity of the data analysis results. It is important to note that depression datasets generally exhibit some common issues, primarily inconsistencies in data collection methods and standards. These inconsistencies often arise from ethical and privacy concerns, leading to the inaccessibility of many datasets. Such issues are particularly prominent in the use of depression datasets and may affect the generalizability of research findings. Therefore, we have included this comparative explanation to help readers better understand the applicability and limitations of these datasets. T, Text; S, Emotional; E, Eyes; D, Depression Specific; U, User Specific; H, Head; A, Audio; V, Visual; I, Image; F, Landmark.

Bold indicates the maximum value of the indicator results in this experiment group.

Although AI-based depression diagnostic methods have improved diagnostic accuracy and objectivity, they still exhibit limitations or weaknesses in interpretability. This is primarily because classical machine learning algorithms and deep learning models have complex structures that involve multiple layers of non-linear transformations during data processing, making it difficult for humans to understand the decision-making process of the model. These models are often referred to as “black boxes” due to their lack of transparency in internal decision-making. This means that while these models can provide highly accurate predictions, their mechanisms and reasons for specific diagnoses are not easily understood. In clinical practice, doctors need to be accountable for diagnostic and treatment decisions. Interpretability ensures that doctors can clearly understand and explain each decision-making process. If the decision-making process of a model is opaque, doctors may question its reliability, and a lack of transparency can lead to unforeseen errors or biases, increasing the risk in clinical decision-making. Additionally, clinical depression diagnoses generally follow a broadly unified clinical diagnostic standard, which these models and methods often do not consider.

To address these issues, we combined the powerful and universal knowledge foundation of the LLMs with clinical diagnostic standards based on scale methods to propose the use of a new generation of depression auxiliary diagnosis method - “LLM as an AI agent for auxiliary diagnosis”. Our approach employs locally interpretable models in the decision-making process. By providing textual descriptions of intermediate outputs and decisions, doctors can verify the accuracy and reliability of the method. Moreover, by adhering to the diagnostic standards, we can improve the efficiency, accuracy, and interpretability of diagnosis.

## 4 Large language model as an AI agent for auxiliary diagnosis

### 4.1 The applications of LLM in the medical field

With the recent release of ChatGPT, some researchers have observed the powerful versatility and emergent potential of big language models, and have also begun to explore the possibility of introducing LLM into the medical field. Among them, in the medical question and answer tasks, Singhal et al. proposed Med-Palm, which achieved an accuracy rate of 67.2% in the medical question and answer task (98). Subsequently, based on this model and some optimization strategies, Med-PaLM2 was proposed (99), which improved its accuracy by 19%, reaching an astonishing 86.5%. Under the tasks of Computed Tomography (CT) and X-ray image description, Microsoft’s LLaVA-Med (100) can infer the pathological condition of patients based on the input image and generate questions and answers about the image. In terms of medical consultation, ChatDoctor (101) can understand medical terminology, procedures, and diagnostic situations. Patients can interact with the ChatDoctor model through chat interfaces and ask questions about health, symptoms, or medical conditions.

This type of LLM related to the medical field has undergone massive data training and fine-tuning, and can efficiently and accurately complete tasks such as medical consultation and medical image description. Meanwhile, due to the fact that the model output is a description of the content of the text, it greatly enhances the interpretability of the model output; It is also possible to verify the reliability of models or methods based on text output. Therefore, combining the automated diagnostic goals of depression with LLM will be a new direction for assisting diagnosis of depression.

### 4.2 AI agent for auxiliary diagnosis

The emergent abilities which integrated with the medical knowledge of the LLMs have shown us more possibilities for automated diagnosis of depression. But unlike simple medical knowledge Q&A tasks, the diagnosis of depression is the result of a comprehensive judgment of multiple aspects of information, including visual, language content, speech, and other behavioral characteristics, as well as external and internal factors such as personal condition, family situation, and indicator data. In addition, the diagnosis of depression also needs to follow medical clinical diagnostic standards in order to accurately identify the user’s depression status. Therefore, the main purpose of depression diagnosis research is to construct a depression diagnosis method or system which integrates multi-source heterogeneous information, follows clinical diagnostic standards, and has interpretability. Using the LLM as a hub could effectively meet the above requirements.

**Figure 7:**
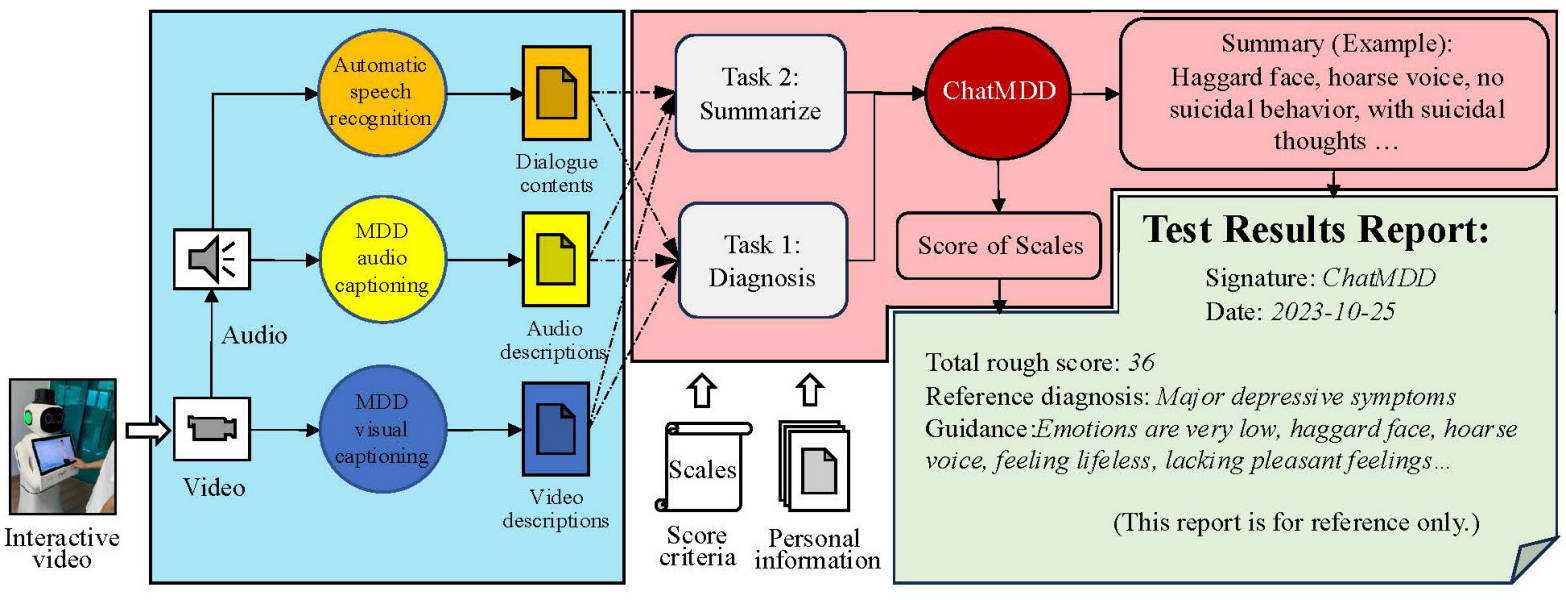
The proposed framework for the fourth-generation depression assistance and diagnostic system. The entire system framework is divided into three parts. The first part involves extracting features from language, speech, and visual modalities using different basic models. The second part involves constructing cue templates based on features and diagnostic criteria through textual descriptions, followed by reasoning through the ChatMDD model. The third part involves formatting the results report.

In the AI agent method, we propose to use different foundation models to extract modal features and provide textual descriptions to enhance feature extraction ability and interpretability. This method also combines the powerful text understanding ability of the LLMs, integrates multi-source heterogeneous information, and achieves objective diagnosis from multiple dimensions. During the scale evaluation process, diagnostic criteria have been introduced to align with practical clinical diagnostic objectives. The proposed framework for the fourth-generation depression assistance and diagnostic system is shown in **Figure 7**.

In this system, we first combine the diagnostic process with the advantages of clinical scale evaluation methods through the interactions of the virtual doctors and interviewees, and finally evaluate the scale through LLM. In the practical research process of the method, we constructed a dialogue transcription model, two foundation feature extraction models (MDD audio model and MDD video model), to describe speech and visual characteristics. We also constructed an LLM-based ChatMDD (Chat Major Depressive Disorder) model for the diagnosis of depression and summarizing personal information. Based on the evaluation process and diagnostic criteria of the scale, the goal of automated diagnosis of depression is achieved. Implementing end-to-end interaction outside the entire system can improve diagnostic efficiency and reduce costs. By integrating multi-source heterogeneous information and the interaction of multiple large-scale models within the system, the situation of respondents can be analyzed from multiple dimensions. Besides, clinical diagnostic criteria were introduced to provide more accurate diagnostic support. The diagnostic results and all details are intuitively expressed in textual form, making the entire system highly interpretable and thus convincing the doctors and respondents with the validity of the diagnostic results. The methodology using the ChatMDD model is compared with other models and previous approaches reported in **Table 3**.

**Table 3:** Comparison of ChatMDD with other models.

Method	Technology Base	Feature Processing	Objectivity	Diagnostic Standard	Interpretability	Scalability
Scale-based evaluation	Scale evaluation & expertise	Standardized questionnaires	Weak	Yes	Yes	No
The first-generation diagnostic methods	Classical machine learning algorithms	Manual feature extraction	Medium	No	Medium	No
The second-generation diagnostic methods	Deep learning models	Automated feature extraction	Medium	No	No	No
The third-generation diagnostic methods	ML + DL advantages	Mixed model for feature extraction	Medium	No	Weak	No
ChatMDD for auxiliary diagnosis	LLMs	Multi-modal data processing and analysis	Yes	Yes	Yes	Yes

Technology Base refers to the core technology underlying each method, such as scale-based evaluation or machine learning algorithms. Feature Processing describes how each method handles data, ranging from manual to automated processing. Objectivity is standardized as “No/Weak/Medium/Yes” to reflect the reliance on automated processing versus subjective judgment. Diagnostic Standard is simplified to “Yes/No” indicating whether the method has a clear diagnostic standard. Interpretability uses “Yes/Weak/No” to show how easily the model’s outputs can be understood. Finally, Scalability is presented as “Yes/No” indicating the method’s adaptability to varying data sizes and complexity levels. Additionally, the table does not suggest that LLM-based methods will replace all previous approaches; each method has its own suitable application scenarios. Scale-based evaluation is appropriate for traditional clinical assessments; methods based on classical machine learning algorithms are suitable for small to medium-scale data analysis and semi-automated processing; deep learning models are ideal for large-scale data analysis and automation; the third-generation hybrid models are well-suited for complex multi-source data analysis; and using LLMs as auxiliary diagnostic agents is most effective in high-demand clinical settings requiring high interpretability.

#### 4.2.1 Feature extraction

**Figure 8:**
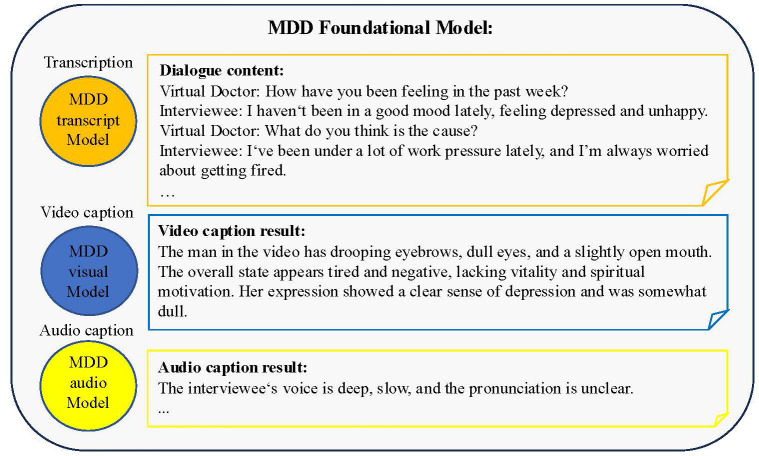
Example illustration of the feature texturization results for the foundational model of MDD. The dialogue content includes interviews between virtual doctors and interviewees, and the questions involved are designed based on diagnostic criteria. The result of audio and video caption is a textual description of speech and visual features.

The process of evaluating the scale was implemented in the form of muti-turns conversations with a virtual visual agent, during which we collected interview videos. After the MDD oriented conversion, the audio corresponding to the scale question can also be separated. By inputting the audio and video data into the foundation models of different modalities, the text descriptions of different modalities can be obtained. The text description results of the foundation model implementation features are shown in **Figure 8**.

For feature extraction methods and fusion techniques of different modalities, our approach diverges from previous approaches that represented features numerically. Instead, we adopted new audio captioner and visual captioner models to describe individual features textually. These textual descriptions were then integrated using a prompt template, which served as the input for LLMs. This method offers superior interpretability and scalability compared to the earlier generations of fusion techniques.

Specifically, the dialogue transcription model can more accurately transcribe complex professional terminology and rare disease types. The MDD audio model can analyze the audio information of the interviewee, capture the audio characteristics related to depression, and express it in the form of text, such as the interviewee’s voice sounding somewhat depressed and low, the volume is low, and the speech is ambiguous. Consistent with the design concept of the language model, the MDD visual model can capture the visual characteristics of respondents, such as common visual features such as dullness, depression, desolation, and eye avoidance, as well as other features that cannot be intuitively detected such as eye movement changes, head posture, and movement unit changes, and express them in text. ChatMDD, through training on massive data and fine-tuning on depression datasets, can not only capture the language content information of interviewees, but also integrate other heterogeneous information such as personal information, medical knowledge, clinical diagnostic standards, audio features, visual features, etc., to assist in depression assessment, summarize interviewee information, and provide clinical guidance for reference. In addition, this architecture can be easily extended to other modalities in subsequent researches, such as electrophysiological foundation models, EEG foundation models, etc.

#### 4.2.2 Prompt template

**Figure 9:**
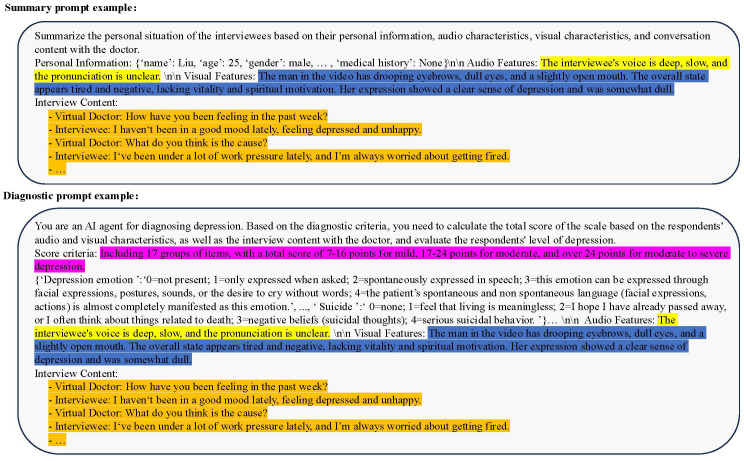
Prompt Template Example: Summary Prompt Template (above), Diagnostic Prompt Template (below). The summary prompt template consists of four parts: personal basic information, audio features, visual features, and interview content. The diagnostic prompt template has changed personal basic information to diagnostic criteria and introduced role and task descriptions.

Integrating multi-source heterogeneous information as input to the ChatMDD also requires constructing appropriate prompt templates. In the above system, we constructed two different prompt templates to summarize the personal information of the respondents and use for depression assessment. In the summarized prompt template, we provide a task description and relevant information, allowing the ChatMDD model to automatically analyze the content and ultimately generate task results. In the diagnosis prompt template, we prompt the model to assume the role of a “depression diagnosis agent” to stimulate its professional capabilities. Additionally, we introduce diagnostic criteria to enhance the reliability of the model’s assessments. The content of the Prompt template is shown in **Figure 9**. It should be noted that the content marked with colors is not complete, and the above is only for the convenience of understanding the construction method of the prompt template. In practical applications, suitable prompt templates can be constructed based on the data information needed.

#### 4.2.3 Diagnosis method and the report of ChatMDD

The diagnostic approach of ChatMDD comprises two methods. The first method involves analyzing all scale indicators at once, suitable for scales with fewer indicators. In this method, the inference process yields the final result in a single step, but the drawback is that the model requires high ability. The second method integrates all information and cyclically evaluates each indicator. Compared to the one-time approach, this method may yield more precise diagnostic results but requires more time for assessment.

**Figure 10:**
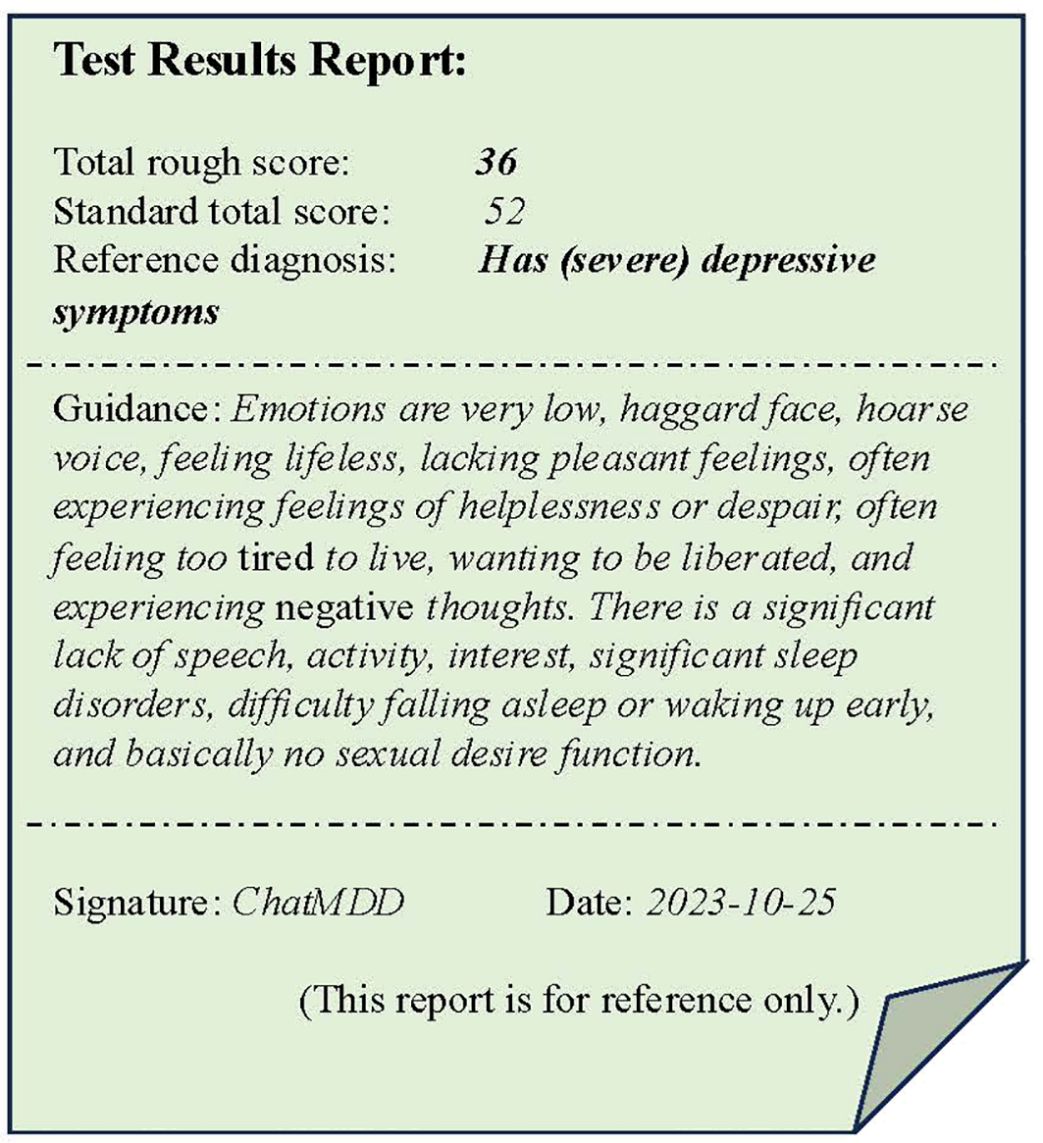
A example of the test results reports. The test result report includes the total score and diagnostic results of the evaluation, as well as a summary of personal situations and some reference guidance.

**Figure 11:**
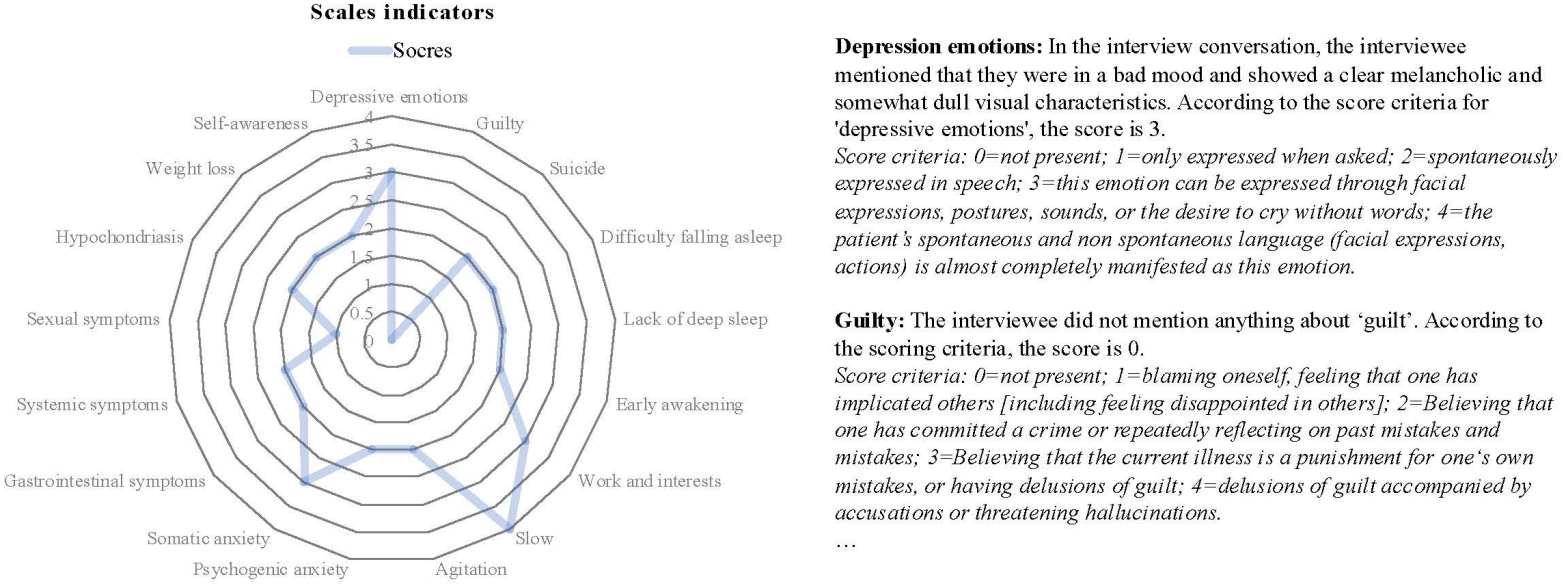
Radar chart of scale indicators’ results (left), along with the assessment outcomes and diagnostic criteria for each indicator (right).

In our system, the final diagnostic result obtained through the above process is shown in **Figure 10**. The ChatMDD model was used to combine all information, and each score was obtained based on the diagnostic criteria. The final score and diagnostic result were obtained. And for each score, we also provide criteria and criteria for judgment to enhance the interpretability of the model output, as shown in **Figure 11**.

#### 4.2.4 Ethics, limitations, and challenges

When deploying AI models for diagnosing mental health conditions such as depression, several key ethical issues must be considered. Firstly, privacy and data protection are crucial given the sensitivity of mental health data. It is essential to ensure data anonymization to prevent patient identity disclosure. Secondly, informed consent is required; patients should be fully aware of how their data will be used and the associated risks. Additionally, the AI model’s working principles, decision bases, and limitations must be transparently communicated to both patients and healthcare professionals.

However, it is important to acknowledge that there are still some limitations to this approach that need further improvement. For instance, due to the variable clinical manifestations of depression, LLMs require extensive and diverse datasets for training and fine-tuning to ensure accurate identification of a wide range of symptoms. Additionally, challenges associated with dataset quality and bias handling, such as demographic imbalances, should be addressed to improve model generalizability and performance. These issues, along with a comprehensive comparison of ChatMDD and other state-of-the-art models in terms of accuracy, explainability, and clinical applicability, will be explored in future studies. Furthermore, clinical validation is the biggest barrier to the practical implementation of AI models in real-world clinical settings. Overcoming this challenge would require large-scale clinical trials and validation, which is something we have been actively working on. Only through thorough clinical validation can we ensure the effectiveness and reproducibility of these models in real-world scenarios, thereby facilitating their widespread adoption in clinical practice.

## 5 Summary

In this paper, we first introduced a scale-based depression diagnosis method, systematically reviewed the research of AI technology in depression diagnosis, and pointed out that the comprehensive, objective, and interpretable results are the problems that need to be addressed in the current depression diagnosis research. However, with the development of LLM technology, many issues identified in previous studies could be addressed by applying it to depression diagnosis. Finally, we propose a framework for the fourth-generation depression auxiliary diagnosis system based on the ChatMDD model and make some further attempts in this area. The new method of AI agent has enormous potentials, but it also faces the following two challenges: (1) high-quality training data is critical for improving the performance of LLM, and the collection of depression patient data is a very difficult and time-consuming task; and (2) the current method of AI agent is to use LLM to process the text data converted from the foundation model. Therefore, by constructing a multimodal LLM as the agent, it will be more in line with the idea of multi-dimensional comprehensive diagnosis.
